# The quality and reliability of short videos about muscle strain on Bilibili and TikTok: Cross-sectional study

**DOI:** 10.1097/MD.0000000000051099

**Published:** 2026-10-16

**Authors:** Wenqian Du, Cheng Chen, Xiaolin Yao, Wenling Gou, Yuwen Shangguan, Xiaobo Xu, Taojing Zhuang

**Affiliations:** a Graduate School, Harbin Sport University, Harbin, China; b Institute of Sports Humanities and Society, Harbin Sport University, Harbin, China; c SanTESiH Laboratory, Faculty of Sports Sciences and Techniques, University of Montpellier, Montpellier, France; d Department of Exercise Physiology, Kunsan National University, Kunsan, South Korea; e Department of Orthopaedics, Changzhou Maternal and Child Health Care Hospital, Changzhou Medical Center, Nanjing Medical University, Changzhou, China; f Department of Orthopaedics, Changzhou Maternal and Child Health Care Hospital, Changzhou Medical Center, Nanjing Medical University, Changzhou, China.

**Keywords:** Bilibili, information quality, muscle strain, short videos, TikTok

## Abstract

Muscle strain-related information is extensively shared on short video platforms, yet the quality and reliability of such content remain unassessed. This study aimed to evaluate the quality and reliability of muscle strain videos on Bilibili and TikTok. A total of 157 eligible videos were assessed using the Global Quality Score (GQS) and modified DISCERN (mDISCERN) scales. Videos were graded as high (GQS 4 to 5, mDISCERN 4 to 5), medium (GQS 3, mDISCERN 3) or low (GQS 1 to 2, mDISCERN 0–2) based on information completeness, logicality, source reliability, and objectivity. Bilibili’s video quality was predominantly medium to high, while TikTok’s quality showed polarization with more medium and low-scoring content. Professional institution-generated videos and scientific introduction-themed content achieved the highest scores; therapeutic content accounted for 82.8% of all videos but was mostly low to medium in quality, and causal content only made up 2.5%. TikTok had significantly higher user interaction metrics than Bilibili (all *P* < .05), and professional individual creators had higher video sharing volumes than professional institutions (*P* = .034). GQS was strongly positively correlated with mDISCERN (*R* = 0.86, *P* < .001); all interaction indicators were highly positively correlated (*R* ≥ 0.81, *P* < .001), and no significant correlation was found between interaction metrics and content quality (e.g., correlation coefficient between GQS and likes = 0.18, *P* > .05). Bilibili demonstrated a better overall quality of muscle strain videos compared to TikTok, which exhibited a more polarized quality distribution with a higher proportion of low-quality content. TikTok showed significantly higher user interactivity, but engagement metrics were not associated with content quality. Both platforms presented an unbalanced topic supply, with an overrepresentation of therapeutic content and a severe scarcity of causal content. These findings highlight the need for optimizing the ecosystem of health popular science short videos, providing targeted references for the public, platforms, and professionals to improve the quality and reliability of muscle strain information online.

## 1. Introduction

Muscle strain is an indirect injury caused by excessive force or stress on the muscles, which often occurs when strong contraction is accompanied by forced lengthening of the muscles, and often involves the tendon junctions of specific muscles such as the adductor longus muscle and rectus femoris muscle, manifested as tissue rupture, fluid accumulation, and edema and inflammation.^[1]^ Mild strains present with slight edema and discomfort, with no meaningful reduction in muscle strength or restriction in activity; moderate strains show markedly impaired muscle function, possible tissue defects at the injury site, and hematomas and ecchymosis may appear within 2 to 3 days.^[2]^ Severe muscle strain is characterized by almost complete loss of muscle function, accompanied by severe pain, extensive ecchymosis, and significant structural defects in the muscle tissue.^[3]^ Specific manifestations include sudden calf pain and edema in gastrocnemius muscle strain, and knee pain with weakness in knee extension in quadriceps strain.^[4]^

Muscle strain has a very high incidence in the general population, and relevant epidemiological data show that about 80% of muscle strain cases occur in daily work, fitness exercise, or noncompetitive sports.^[5]^ Compared with athletes, who can rely on professional coaches, team doctors, and sports rehabilitation therapists to obtain standardized injury assessment, treatment, and rehabilitation guidance,^[6]^ habitually active adults often lack such professional support channels.^[7]^ After suffering from muscle strain, this group is more inclined to retrieve fragmented information through social media, evaluate the degree of injury and try home treatment options.^[8]^ However, habitually active adults generally lack the detailed knowledge related to sports injury. (In this study, “habitually active adults” refers to adults who regularly participate in physical activity or exercise but lack professional knowledge of sports injuries.) Unlike authoritative platforms (e.g., CDC official website, professional medical databases) that provide structured, evidence-based information (e.g., typing “muscle strain CDC” directly yields standardized guidelines on causes, treatments, and recovery),^[9]^ short video platforms feature uncurated content mixed with professional advice, personal experience, and even misleading claims with no clear labeling of information sources or peer-review verification. This makes it difficult for habitually active adults to evaluate the rigor and applicability of the information they encounter, increasing the risk of aggravating the injury, prolonging the recovery period, or even inducing chronic pain or secondary injury due to incorrect treatment.^[9]^

For athletes, muscle strain can lead to missed competitions, disrupted training schedules, and reduced performance even after rehabilitation.^[10]^ While nonsteroidal anti-inflammatory drugs (NSAIDs) can alleviate pain in the early stages, they may delay the muscle repair process.^[11]^ However, awareness of muscle strain remains insufficient among the general public and even some medical professionals a gap that hinders the timely intervention of the condition to a certain extent.^[12]^ Therefore, improving public awareness of muscle strain remains an unmet clinical and public health need.

The increasing popularity of social media has become an influential way of knowledge acquisition and dissemination. In recent years, short video, with its unique advantages, has gradually replaced traditional text and become a main form of social media communication^[13]^ (Table 1). Compared with the fragmented information retrieval of search engines such as Google and the nonempirical information generation of artificial intelligence tools such as Chat GPT, the advantages of short video in the field of health science popularization have been confirmed by a number of studies^[14]^: Firstly, visual presentation can intuitively display practical contents such as symptom recognition and rehabilitation training of muscle strain through dynamic demonstration, and reduce the understanding threshold of professional knowledge, which is especially suitable for the theme of sports injury that needs standardized guidance^[15]^; Secondly, the algorithm can quickly reach the target population who regularly participate in physical activity or exercise, and improve the dissemination efficiency of popular science information^[16]^; thirdly, 2-way interaction supports users to ask questions in the comment area and creators to answer questions in real time, enhancing the pertinence of knowledge acquisition and user participation.^[17]^ In China, TikTok and Bilibili are the most popular video social platforms, with hundreds of millions of users.^[18]^ Users can easily create and share short videos, and interact with each other through comments, likes and other functions, making the platform extremely popular.^[19]^ However, the platform lacks a filtering mechanism,^[20]^ the quality and reliability of video content are poor, and some misleading information still exists.^[21]^ Although a large number of short videos on TikTok and Bilibili have emerged on the topic of muscle strain, which can provide ordinary patients with easy-to-understand health knowledge and help improve the public ‘s awareness of the injury, the scientific rigor of such videos has not been systematically evaluated.

**Table 1:** Main types of social media and their roles in health information dissemination.

Media type	Example platforms	Core function in health communication
Short-video platforms	Bilibili, TikTok	Visualize health knowledge via dynamic content
Text-based platforms	Weibo, WeChat	Disseminate text-based health information
Search engines	Google, Baidu	Provide fragmented health information retrieval
AI tools	ChatGPT, Wenxin Yiyan	Generate non-empirical health information

This study uses a comprehensive quality assessment method to objectively evaluate the quality of related videos and provide a basis for formulating content quality improvement strategies.^[22]^ Mature tools such as the Global Quality Score (GQS) and Modified DISCERN (mDISCERN) have been widely used to evaluate the integrity, quality and reliability of short videos on various health-related topics on social media, including chronic diseases such as diabetes and stroke, acute musculoskeletal conditions or injuries such as muscle strain and sports injury, and infectious diseases such as COVID-19.^[23,24]^ This study will use these 2 tools to evaluate the quality and reliability of short video related to muscle strain, and analyze the correlation between video features (source, type, interaction index) and quality score, so as to provide evidence-based guidance for patients and their families to identify reliable online health information and assist health decision-making.

## 2. Materials and methods

### 2.1. Ethical considerations

This study was conducted through the analysis of publicly available video materials and did not involve clinical data, human samples, or laboratory animals. No personally identifiable information was collected from the video during the data extraction process, and no interaction was made with the video uploader or viewer throughout the study. Therefore, ethical approval is not required for this study. In addition, all data is anonymized during the analysis process, and no individual user, video, or screenshot information can be identified in this paper or its supplementary materials.

### 2.2. Search strategy and data extraction

To minimize selection bias introduced by newly uploaded videos, video retrieval was conducted on TikTok and Bilibili platforms on October 15, 2025, using “muscle strain” as the core search term. Videos published within 7 days and content bearing advertising labels were excluded during initial screening.

Site-specific terms such as “calf strain” and “hamstring strain” were intentionally not employed in the search strategy, based on three methodological considerations. First, this approach aligns with the information-seeking behavior of the target population: this study focuses on habitually active adults, who typically lack specialized knowledge of sports injuries and tend to use the general term “muscle strain” when searching. Second, using general keywords improves retrieval coverage: content labeling on short video platforms tends to be colloquial and and generalized, and most site-specific videos include “muscle strain” in their titles or tags, thus avoiding omissions caused by inconsistent labeling. Third, this strategy ensures the feasibility of subsequent stratified analysis by enabling clear formulation of inclusion and exclusion criteria while allowing reclassification of injury sites presented in the videos.

#### 2.2.1. Inclusion criteria

Videos were considered eligible if they met the following requirements: the content addressed acute skeletal muscle strain resulting from excessive force, covering common injury sites including calf, posterior thigh, and lumbodorsal muscles; the content type included topics directly related to muscle strain, such as symptom identification, emergency management, rehabilitation training, and preventive measures; and the content was clearly associated with physical activity or exercise contexts, or the core information was applicable to injury prevention and rehabilitation needs of physically active populations.

#### 2.2.2. Exclusion criteria

Videos were excluded if they met any of the following conditions: the content focused on non-tensile musculoskeletal injuries such as muscle contusion, laceration, and tendinitis; the video theme was not directly related to muscle strain, with only incidental mention of relevant terminology; the content represented obvious duplicate videos; or and the content focused on contexts unrelated to physical activity or exercise.

To eliminate interference from previous search histories and platform personalized recommendation algorithms, new user accounts were created on both platforms specifically for data collection. Following keyword searches, the first 100 results from each platform’s default ranking were retrieved and systematically screened according to the criteria specified above. Ultimately, 157 eligible videos were included in the final analysis, comprising 87 videos from TikTok and 70 videos from Bilibili.

Comprehensive metadata was extracted for all included videos, encompassing title, uploader identity, user engagement metrics (likes, favorites, comments, shares), content category, creator source, video duration, and publication date. Three analytical dimensions were established based on these extracted variables.

#### 2.2.3. Platform comparison of user interaction

Between-platform comparisons were performed to examine differences in user interaction indicators (likes, favorites, comments, and shares) between TikTok and Bilibili.

#### 2.2.4. Creator source classification and propagation characteristics

Variations in video dissemination characteristics were analyzed across different creator sources. Creator background plays a critical role in shaping the quality and reliability of muscle strain-related health information, as professional qualifications and practical experience directly influence the accuracy and clinical applicability of content regarding patient treatment and rehabilitation. Four creator categories were defined using standardized classification criteria. Professional individuals were defined as rehabilitation physicians holding medical or rehabilitation-related professional qualification certificates with specialized expertise in sports injury diagnosis and management, as well as platform- or sports authority-certified bloggers with a long-term focus on sports medicine and rehabilitation domains. Professional institutions were defined as industry-certified entities with standardized organizational structures whose content output strictly adheres to professional standards and evidence-based principles, including official accounts of rehabilitation medicine departments in tertiary hospitals and specialized sports rehabilitation research institutions. Nonprofessional individuals were defined as individuals without relevant professional certification who share experiences based on personal muscle strain history during physical activity, sports-related master’s or doctoral graduate students with theoretical knowledge but lacking official clinical or content creation certification, and uncertified bloggers sharing muscle strain content based on personal interests and amateur experience without platform or authority verification. Nonprofessional institutions were defined as entities holding industry qualifications in medical, rehabilitation, or sports fields but lacking official short video platform certification, such as uncertified rehabilitation studios and specialized sports clubs.

#### 2.2.5. Video theme distribution and interactive performance

The proportional distribution of video themes was analyzed, and interactive performance was compared across different content categories. Video themes were classified according to the following criteria. Causes referred to content explaining the inducing factors and pathological mechanisms of muscle strain. Scientific introduction referred to content systematically elaborating on the classification, diagnostic criteria, and clinical features of muscle strain. Judgment category referred to content guiding identification and severity assessment of muscle strain conditions.

### 2.3. Video evaluation

In this study, the mDISCERN scale and the GQS, two widely used standardized tools, were used to systematically evaluate the information quality and content reliability of the included videos.^[23]^ The improved version of DISCERN is one of the most widely used tools for assessing the quality of health information^[25]^; its effectiveness has been fully validated in health information quality assessment research of short video platforms such as TikTok.^[26]^ The scale is quantified from 5 dimensions: clarity, relevance, traceability, robustness, and fairness. A set of 5 questions is employed, with each question answered in a “yes/no” binary classification. “Yes” is counted as 1 point, “no” is counted as 0 point, and the full score is 5 points. The GQS is a widely used 5-point scale that has been validated for assessing health information quality across multiple digital platforms.^[27]^ GQS contains 5 items with scores ranging from 1 to 5. The higher the score, the better the quality.^[24]^ The specific scoring criteria are shown in Table 2 and Table 3.

**Table 2:** The Global Quality Score (GQS) quality criteria.

Item features	Points
Poor quality; poor flow of the videos; most information missing; not at all useful for patients	1
Generally poor quality; some information listed, but many important topics are missing; of very limited use to patients	2
Moderate quality; suboptimal flow; some important topics adequately discussed, but other information poorly discussed; somewhat useful for patients	3
Good quality and generally good flow; most of the relevant information is listed, but some topics are not covered; useful for patients	4
Excellent quality and flow; very useful for patients	5

**Table 3:** The Modified DISCERN (mDISCERN) quality criteria.

Reliability Score
1. Is the video clear, concise, and understandable?
2. Are valid sources cited?
3. Is the content presented balanced and unbiased?
4. Are additional sources of content listed for patient reference?
5. Are areas of uncertainty mentioned?

All included videos were retrieved, collected, and downloaded by the same researcher. In order to reduce order deviation in the evaluation process, a random number table method is used to randomize the video evaluation order. Subsequently, 2 sports rehabilitation graduate students with a sports medicine research background conducted independent double-blind evaluations and scoring after completing the video theme classification. Before the formal scoring, the 2 evaluators jointly studied the evaluation dimensions, standards, and operating rules of the scale, and ensured the consistency of the evaluation criteria by pre-evaluating 10 noninclusion video calibration scoring strategies. Differences or disputes arising from the evaluation process were adjudicated by a third attending physician with clinical experience in the diagnosis and treatment of sports injuries. The classification results and scoring data of all videos were verified by all researchers. Cohen’s kappa coefficient(Landis JR, Koch GG, 1977) was used to test the inter-rater reliability, and the results showed good consistency (*κ* = 0.75).

### 2.4. Statistical analysis

Descriptive statistical methods were used to summarize continuous variables. Normally distributed data are expressed as mean ± standard deviation (SD), and non-normally distributed data are expressed as median and interquartile range (IQR). Categorical variables are expressed as frequencies and percentages. For comparison between groups, the independent sample *t* test was used for normally distributed variables, and the Mann–Whitney *U* test was used for non-normally distributed variables. The Kruskal-Wallis *H* test was used for comparisons of 3 or more groups, and when the difference was statistically significant, Dunn’s test was further used for 2-by-2 comparisons. For grouped data by platform, creator source, and video theme, the chi-square test or Fisher’s exact probability method was used for categorical variables, and the Mann–Whitney *U* test or Kruskal-Wallis H test was used for continuous interaction indicators. The Spearman rank correlation coefficient was used to assess the correlation between video quality scores GQS vs mDISCERN and interaction metrics (likes, comments, shares, and favorites). A two-sided *P* value of <.05 was considered statistically significant. All statistical analyses were performed in R software (version 4.3.2). Definition added: The term “polarized” in this study refers to a bimodal distribution of scores, where videos are concentrated at either very high or very low values, with few intermediate scores.

## 3. Results

### 3.1. Overview of video screening process

From TikTok and Bilibili, we retrieved and sorted the top 100 videos related to muscle strain. After careful screening, we removed 13 duplicate and irrelevant videos from TikTok and 30 duplicate and irrelevant videos from Bilibili. As a result, 87 suitable videos from TikTok and 70 from Bilibili were selected for subsequent analysis. Ultimately, a comprehensive evaluation was conducted on 157 qualified videos from the TikTok and Bilibili platforms (Fig. 1).

**Figure 1.**
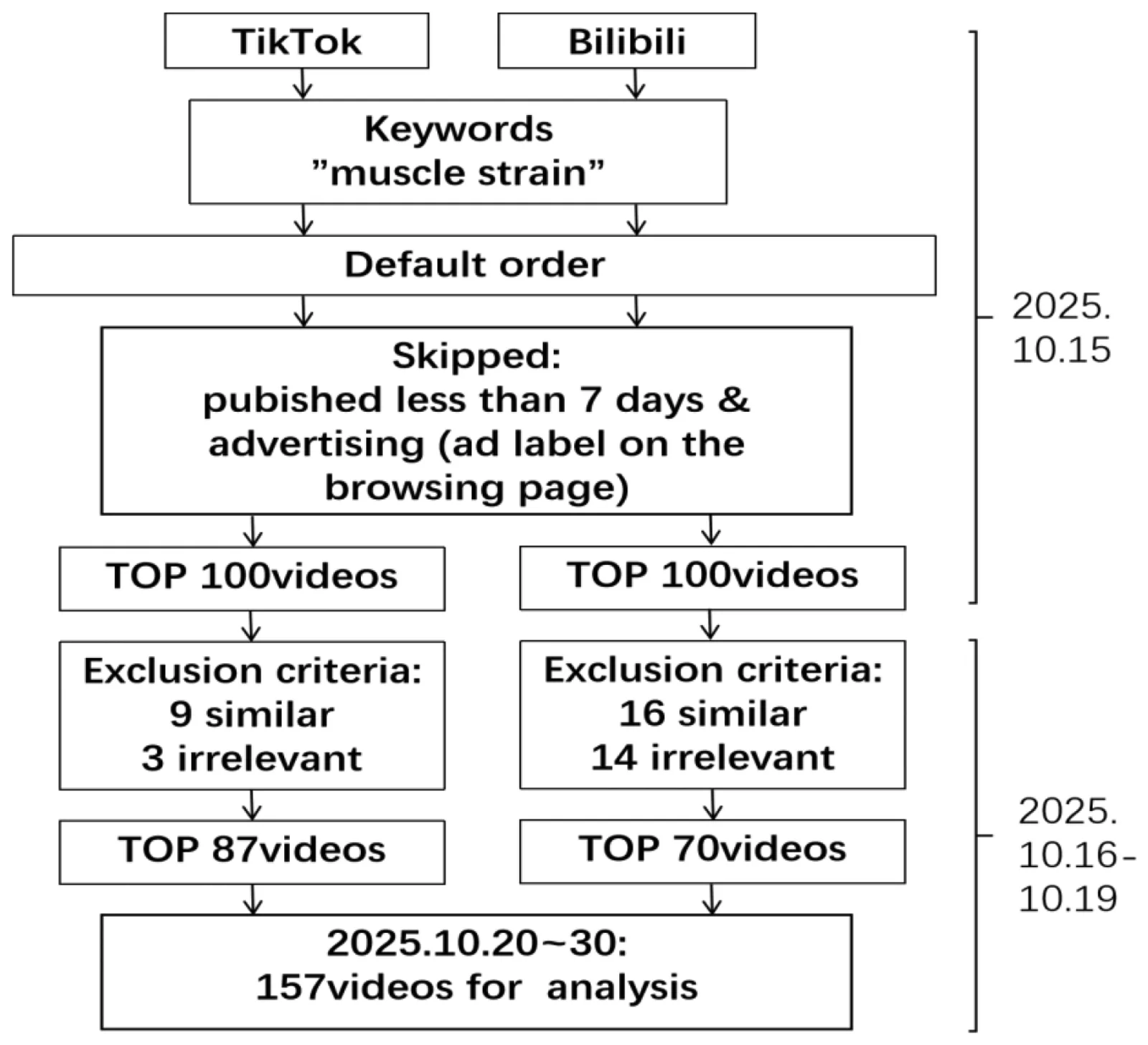
Flowchart of muscle strain video screening and analysis.

### 3.2. Video feature analysis

Comparison of platform interaction metrics revealed that TikTok exhibited significantly higher median values across all engagement indicators compared to Bilibili: likes (813.00 vs 138.00), saves (199.00 vs 119.50), comments (58.00 vs 11.00), and shares (261.00 vs 45.50) (all *P* < .05, Mann–Whitney *U* test, Table 4).

**Table 4:** Differences in user engagement features between TikTok and Bilibili.

Variables	Total (n = 157)	bilibili (n = 70)	TikTok (n = 87)	Statistic	*P*
Likes, M (Q_1_, Q_3_)	321.00 (91.50, 1371.00)	138.00 (31.00, 384.50)	813.00 (263.00, 3124.50)	*Z* = −5.82	<.001
Favorites, M (Q_1_, Q_3_)	171.00 (51.00, 647.00)	119.50 (38.50, 423.50)	199.00 (69.00, 1021.50)	*Z* = −2.48	.013
Comments, M (Q_1_, Q_3_)	25.00 (5.00, 123.00)	11.00 (2.00, 49.00)	58.00 (10.50, 205.00)	*Z* = −4.21	<.001
Shares, M (Q_1_, Q_3_)	96.00 (22.00, 445.00)	45.50 (5.00, 114.50)	261.00 (52.00, 1150.50)	*Z* = −5.68	<.001

*Z*: Mann-Whitney test, χ^2^: Chi-square test, -: Fisher exact, *: Simulated *P* value.

M: Median, Q_1_: 1st Quartile, Q_3_: 3st Quartile.

Analysis by creator source demonstrated that the share count of videos uploaded by professional individuals (244.50 [35.00, 692.50]) was significantly higher than that of professional institutions (77.00 [11.00, 105.00], *P = .034*, Kruskal-Wallis *H* test), while there were no statistically significant differences in like, save, and comment metrics across different sources (all *P* > .05; Table 5, Fig. 2). The topic distribution showed a marked imbalance: treatment methods accounted for the highest proportion (82.8%, 130/157), followed by judgment-related (7.6%, 12/157), scientific introduction (5.7%, 9/157), and cause-related (2.5%, 4/157); however, there were no significant differences in interaction metrics across topics (all *P* > .05, Kruskal-Wallis *H* test; Table 6, Fig. 3).

**Table 5:** Differences in user engagement metrics across creator source categories.

Variables	Total (n = 157)	Nonprofessional individuals (n = 38)	Nonprofessional institutions (n = 60)	Professional individual (n = 50)	Professional Institutions (n = 9)	Statistic	*P*
Likes, M (Q_1_, Q_3_)	327.00 (92.00, 1501.00)	227.00 (42.00,762.00)	308.50 (119.00,1353.25)	437.50 (156.00,2331.00)	198.00 (38.00,813.00)	*χ*^2^ = 4.95#	.175
Favorites, M (Q_1_, Q_3_)	175.00 (51.00, 664.00)	119.50 (28.25,438.25)	240.50 (63.00,746.75)	179.50 (48.50,732.75)	88.00 (14.00,201.00)	*χ*^2^ = 3.93#	.269
Comments, M (Q_1_, Q_3_)	25.00 (5.00, 123.00)	20.00 (4.25,160.75)	29.00 (7.00,100.00)	47.00 (6.00,160.75)	5.00 (2.00,42.00)	*χ*^2^ = 2.93*	.403
Shares, M (Q_1_, Q_3_)	96.00 (22.00, 445.00)	40.00 (5.00,242.50)	92.00 (33.00,431.25)	244.50 (35.00,692.50)	77.00 (11.00,105.00)	*χ*^2^ = 8.65*	.034

M: Median, Q_1_: 1st Quartile, Q_3_: 3st Quartile.

* Kruskal-waills test.

**Table 6:** Differences in user engagement metrics across video theme categories.

Variables	Total (n = 157)	Genesis (n = 6)	Scientific presentation (n = 9)	Judgment (n = 12)	Treatment (n = 130)	Statistic	*P*
Likes, M (Q_1_, Q_3_)	327.00 (92.00, 1501.00)	301.00 (162.50,666.75)	750.00 (34.00,816.00)	640.00 (288.00,1169.75)	315.00 (89.50,1639.25)	*χ*^2^ = 0.57#	.902
Favorites, M (Q_1_, Q_3_)	175.00 (51.00, 664.00)	111.50 (68.50,287.25)	286.00 (51.00,459.00)	120.50 (45.50,307.25)	178.00 (53.00,739.00)	χ^2^ = 0.95*	.812
Comments, M (Q_1_, Q_3_)	25.00 (5.00, 123.00)	16.00 (7.25,87.00)	19.00 (4.00,163.00)	46.00 (4.75,135.50)	25.50 (5.25,110.00)	*χ*^2^ = 0.09*	.993
Shares, M (Q_1_, Q_3_)	96.00 (22.00, 445.00)	112.00 (49.50,257.00)	154.00 (15.00,197.00)	176.00 (33.50,468.75)	90.00 (22.00,451.25)	*χ*^2^ = 0.23*	.972

M: Median, Q1: 1st Quartile, Q3: 3st Quartile.

* : Kruskal-waills test.

**Figure 2.**
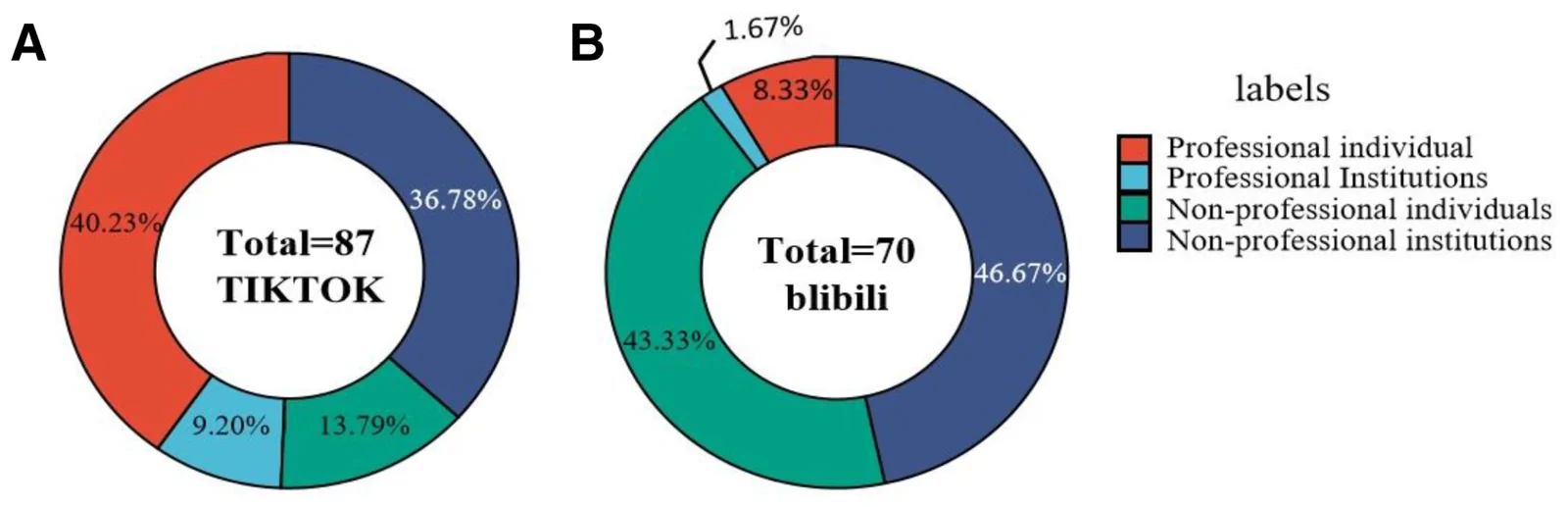
Comparison of the percentage of sources and topics across different platforms. (A) TikTok, (B) Bilibili.

**Figure 3.**
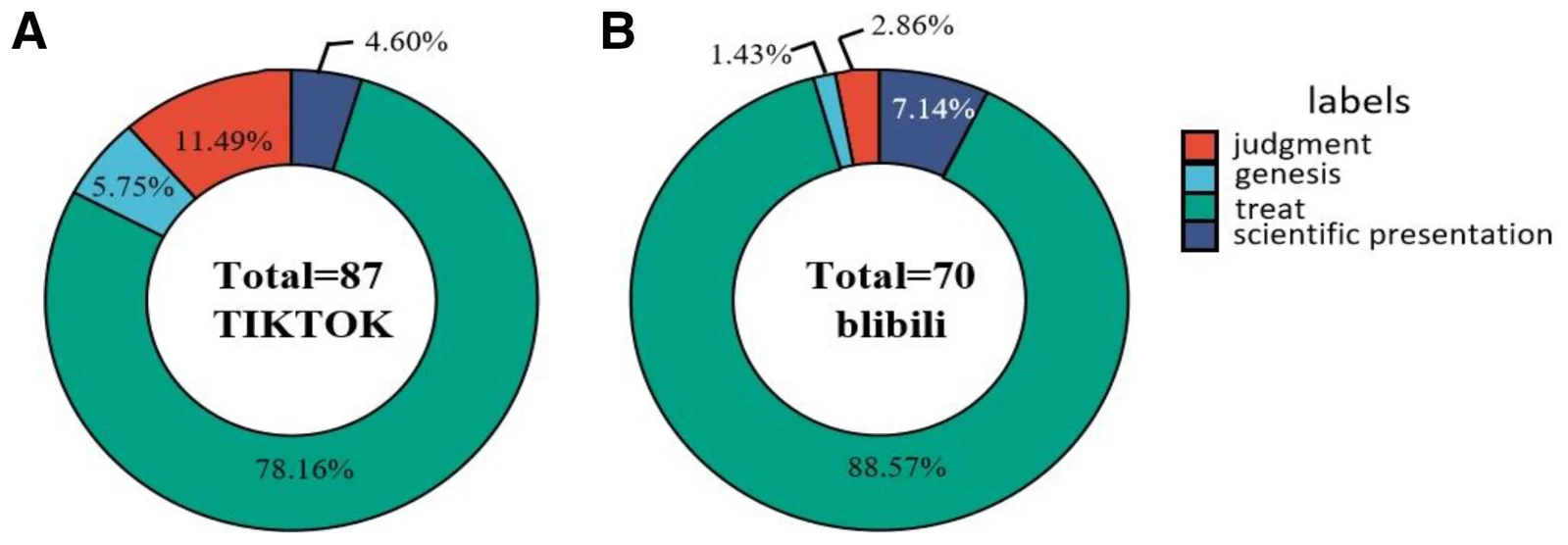
Comparison of topic percentages by platform. (A) TikTok, (B) Bilibili.

### 3.3. Video reliability and quality analysis

The improved version of mDISCERN and the GQS assessment revealed distinct quality distribution patterns for the 2 platforms (Fig. 4, Fig. 5). Full original scoring data, stratified by platform, are provided in Supplemental Appendix 1, Supplemental Digital Content 1 and Supplemental Appendix 2, Supplemental Digital Content 2. Bilibili’s mDISCERN score shows a unimodal distribution with a higher proportion of medium and high scores, and the GQS shows a multimodal distribution with a high proportion of high-quality content. The TikTok mDISCERN scores were bimodal, the GQS was left-skewed, and the proportion of low and medium scores was higher, but there was no significant difference in the overall scores of the 2 platforms (*P* > .05).

**Figure 4.**
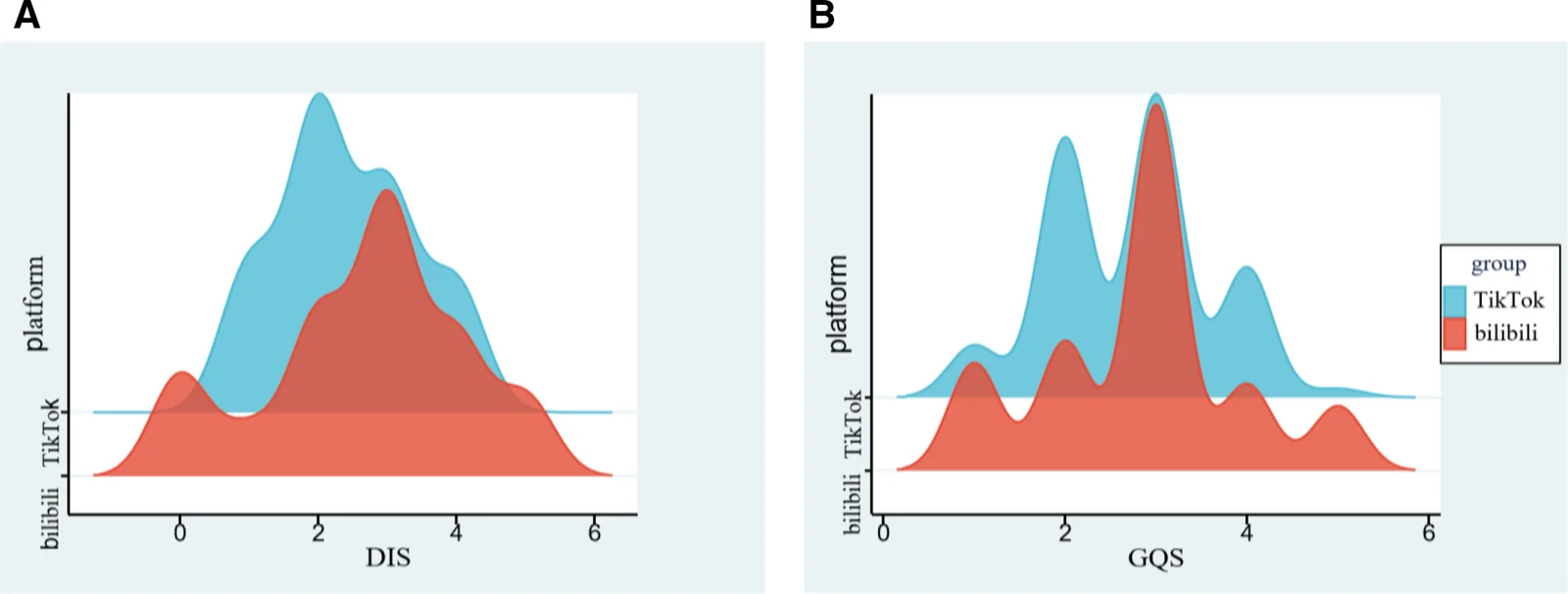
Overall reliability and overall quality score graphs. (A) TikTok, (B) Bilibili. GQS = Global Quality Score.

**Figure 5.**
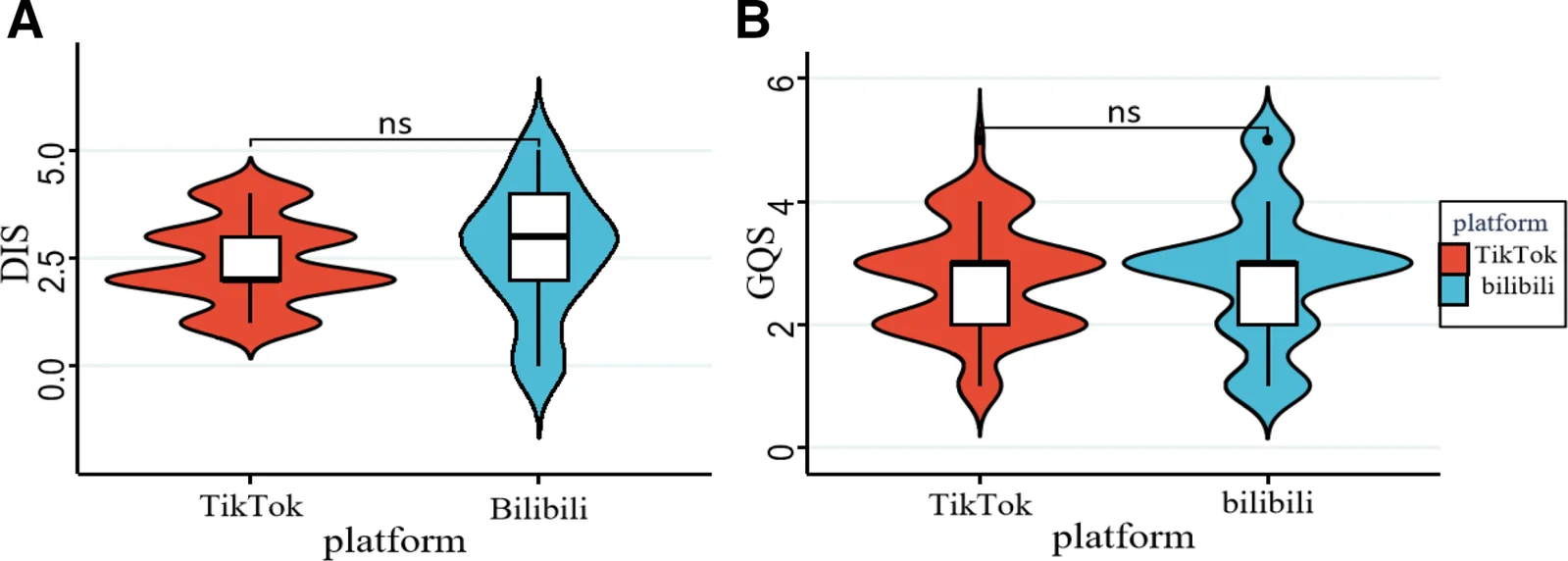
Reliability and quality comparison of different platforms. (A) TikTok, (B) Bilibili. GQS = Global Quality Score.

The source of creators has a significant impact on quality (Fig. 6). Complete scoring data grouped by creator categories are summarized in Supplemental Appendix 1, Supplemental Digital Content 1 and Supplemental Appendix 3, Supplemental Digital Content 3:Professional institutions exhibited the highest scores in both mDISCERN and GQS scores; professional individuals maintained moderate levels; nonprofessional individuals show a bimodal score distribution; the scores of nonprofessional institutions are mostly in the low-to-moderate range. In the theme dimension, the quality and reliability of scientific introduction rank first; the treatment class and the judgment class are at a moderate level; the number of cause-related classes is limited in volume and exhibited substantial variation (Fig. 7). Stratified scores across 4 video themes are listed in Supplemental Appendix 4, Supplemental Digital Content 4.

**Figure 6.**
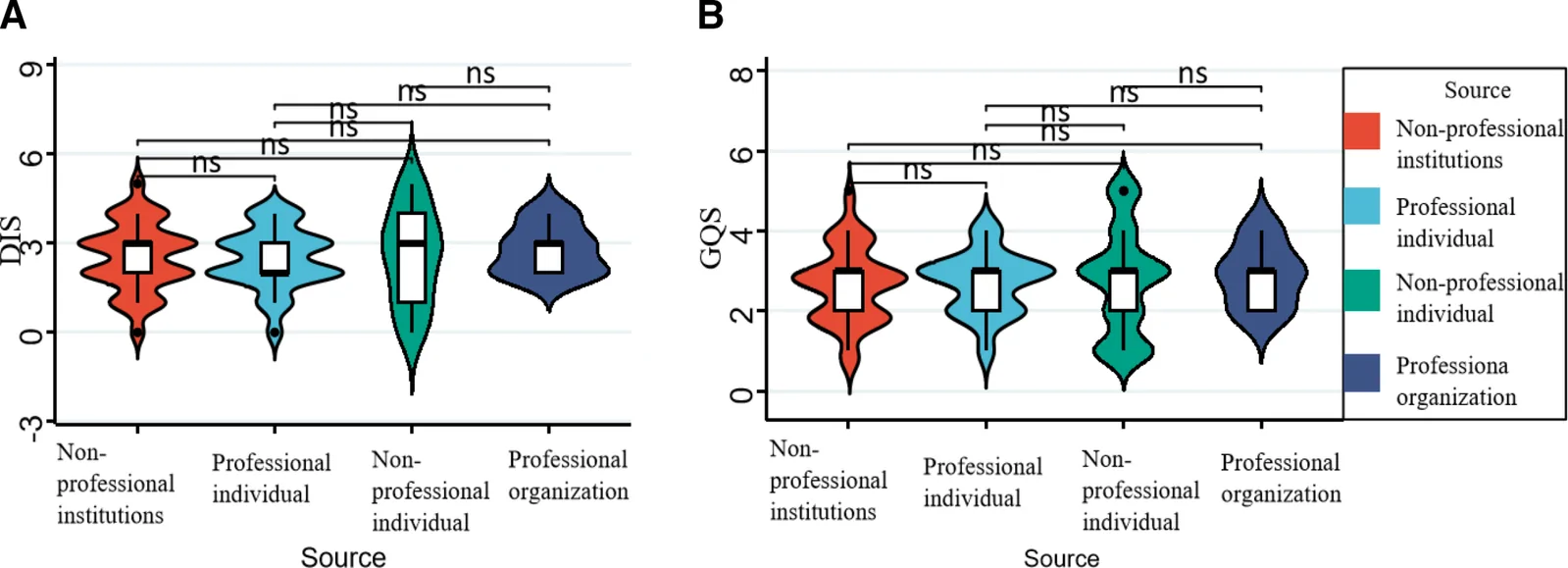
Reliability and quality from different sources. (A) TikTok, (B) Bilibili. GQS = Global Quality Score.

**Figure 7.**
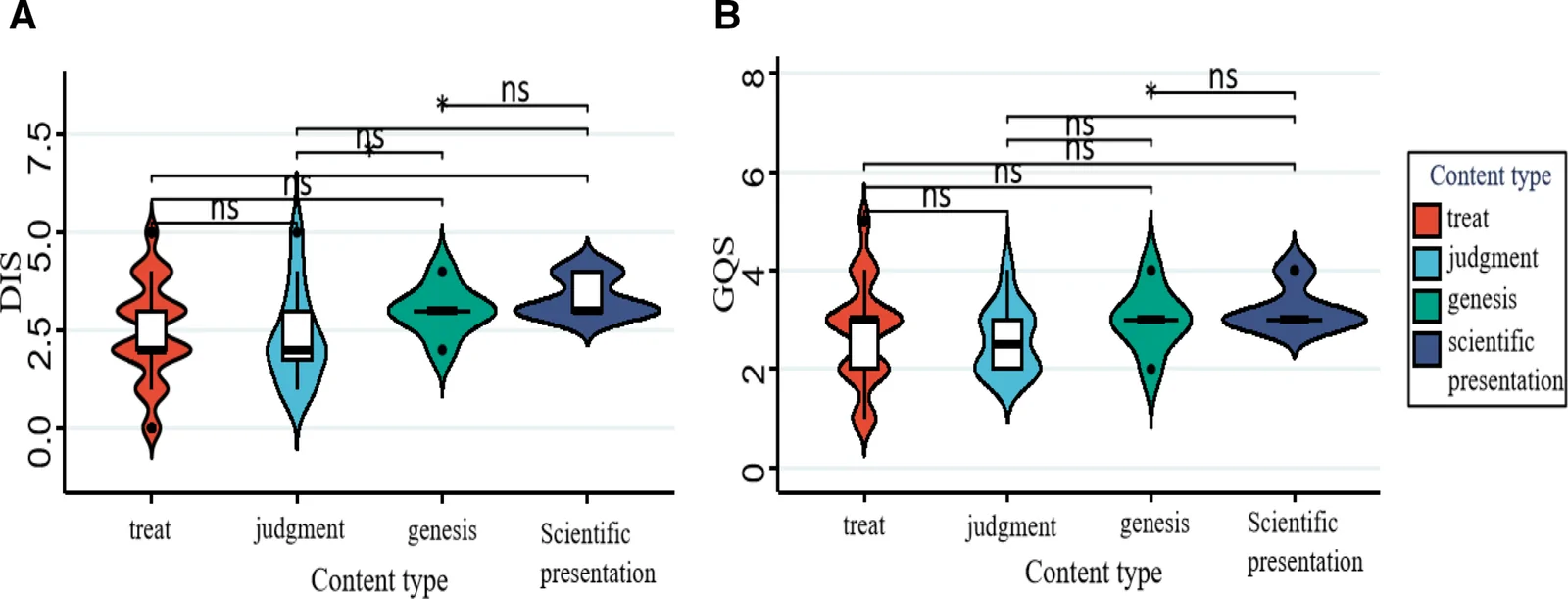
Reliability and quality comparison chart of different topics. (A) TikTok, (B) Bilibili. GQS = Global Quality Score.

### 3.4. Correlation analysis between video quality and user engagement

Correlation analysis revealed 3 distinct patterns between video quality scores and user engagement indicators. First, the GQS score exhibited a strong, significant positive correlation with the mDISCERN score (*R* = 0.86, *P* < .001). This finding indicates good internal consistency between the 2 tools used to evaluate health video quality. Second, likes, favorites, comments, and shares demonstrated strong positive correlations with one another (all *R* ≥ 0.81, *P* < .001). This result reflects the synergistic nature of different user interaction behaviors on short-video platforms. Third, no statistically significant correlation was detected between user engagement metrics and content quality scores. For instance, the correlation coefficient between the GQS score and the number of likes was only 0.18 (*P* > .05, Fig. 8). This outcome suggests that higher public engagement does not correspond to scientifically more reliable muscle strain-related short-video content.

**Figure 8.**
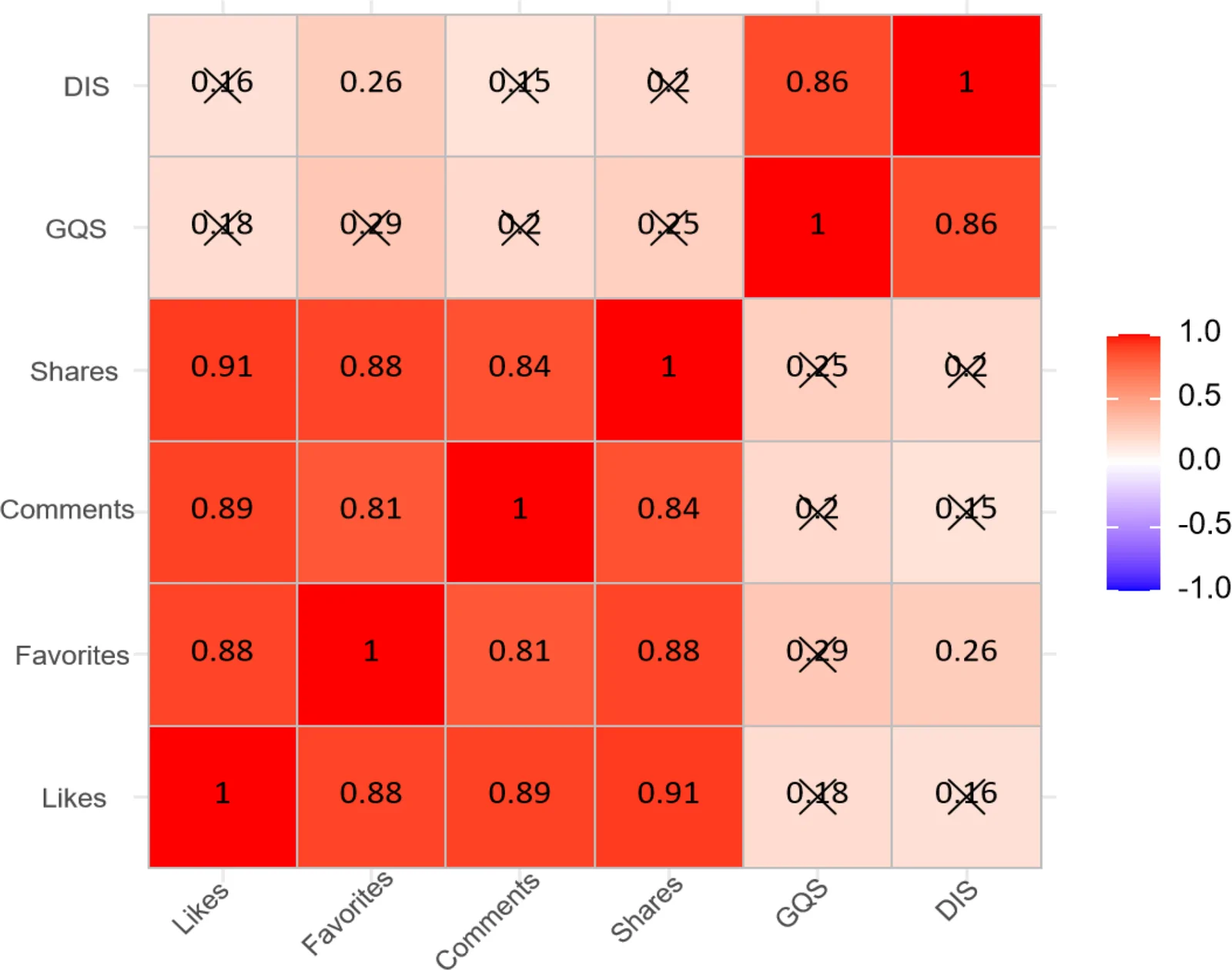
Correlation Heatmap (Spearman). GQS = Global Quality Score.

## 4. Discussion

The purpose of this study is to systematically evaluate the quality and reliability of short videos related to muscle strain on TikTok and Bilibili platforms. We also aim to analyze the source of video creators, topic distribution, user interaction indicators, and quality scores to provide an evidence-based basis for the public to identify reliable health science content and optimize the platform’s science ecology. The main findings show that the video quality distribution of the 2 platforms is significantly different. Bilibili has a higher proportion of high-quality and high-reliability content, while TikTok video quality is polarized and has a higher proportion of medium and low scores. A strong correlation was observed between video quality and reliability, but user engagement is not correlated with professional value. Platform interaction patterns differed significantly, with TikTok demonstrating substantially higher user engagement metrics compared to Bilibili, and professional individual creators achieving higher share volumes than professional institutions. Video theme supply was markedly imbalanced, with treatment-focused videos representing 82.8% of content while etiology-focused content accounted for only 2.5%, indicating a clear gap in health science content structure.

### 4.1. Discussion on interaction indicators

TikTok’s interaction indicators are significantly higher than Bilibili, which is closely related to TikTok’s algorithmic recommendation’s traffic aggregation effect and the “short, flat, and fast” content dissemination model.^[28]^ This mechanism can quickly amplify content exposure and stimulate user interaction. It is worth noting that the volume of professional individual video sharing is significantly higher than that of professional institutions. This may be due to the content of professional individual creators having a greater advantage in scenario-based expression, making it easier to evoke user emotional resonance and dissemination willingness.^[29]^ While professional institutional content may be authoritative, it may reduce dissemination efficiency due to rigid expression forms. There were no differences in interaction indicators across various topics, suggesting that the public’s demand for content related to muscle strains is concentrated on “solving practical problems,” with no significant differentiation in preferences for topic types.^[30]^

### 4.2. Core issues and attribution of video content quality

The core findings of this study reveal 2 major issues in short videos on muscle strains: “imbalanced thematic supply” and “significant quality stratification.” Treatment methods account for 82.8% of the content, while causation accounts for only 2.5%. Scientific introductions are of the highest quality, whereas treatment methods, despite being the most prevalent, are of only medium-low quality. This characteristic aligns with the conclusions of Sun et al.^[19]^ on gallstone videos and Yang et al.^[25]^ on thyroid cancer videos, both demonstrating the common problem of “over representation of popular themes with low quality.” However, the scarcity of causation content is more pronounced in this study He et al^[13]^ found a balanced distribution of causation and treatment themes in videos on inflammatory bowel disease, whereas causation content in this study constitutes <3%. This reflects a more severe neglect of the “prevention-causation” foundational aspects in sports injury science popularization.

The causes of these issues can be analyzed from three complementary perspectives. First, on the creation side, platform traffic orientation leads creators to prefer low-barrier, high-attention topics such as treatment methods, while etiology analysis requires systematic professional knowledge support, creating higher entry barriers for content production.^[31]^ Second, on the demand side, public awareness of muscle strains remains focused on post-injury treatment as an immediate need, with potential demand for prevention and etiology not being fully recognized, thus creating a vicious cycle between demand patterns and creation incentives.^[31]^ Third, on the professional side, scientific introduction content is often produced by professional entities emphasizing evidence-based information, while treatment content involves a more diverse creator base, with some nonprofessional creators oversimplifying professional content to pursue broader audience reach.^[32]^

Another notable finding of this study is the dissociation between user engagement and content quality. Consistent with previous research on temporomandibular disorder videos,^[33]^ user engagement metrics were not associated with professional quality scores,^[34]^ suggesting that high popularity does not necessarily reflect high informational value. Lower-quality videos may achieve greater reach through entertainment or emotional resonance rather than scientific accuracy. This pattern underscores the need to strengthen public media literacy education so that users can better distinguish reliable health information from merely popular content.

### 4.3. Discussion on quality and reliability indicators

The overall professional level in the short video science communication field still has significant room for improvement and has not yet formed platform-specific quality barriers. The quality distribution differences between the 2 platforms essentially reflect platform positioning and user needs: Bilibili users are more inclined to actively seek professional knowledge and have high demands for content rigor, which in turn drives creators to improve quality.^[35]^ Therefore, mDISCERN shows a unimodal distribution with medium-high scores, while GQS exhibits multimodal distribution with prominent high scores. TikTok, on the other hand, focuses on fragmented browsing, with users preferring entertainment and convenience. Creators may simplify content to chase traffic,^[36]^ leading to a bimodal mDISCERN distribution and a left-skewed GQS distribution. This difference aligns with the conclusions of Ma et al.^[37]^ on platform comparisons for pancreatitis videos, both verifying the core principle that “platform ecosystems determine content quality characteristics.”

In the creator dimension, professional institutions have the highest quality due to their standardized, evidence-based creation processes and multi-round review mechanisms^[38]^; professional individuals have moderate quality but stronger communicability, reflecting the “trade-off between professionalism and communicability”^[29]^; nonprofessional individuals show polarization, with some who have a sports background capable of producing high-quality content, suggesting that the “nonprofessional” label does not fully represent content value.^[32]^ In the topic dimension,^[19]^ scientific introduction types rank first due to “logical completeness + sufficient evidence,” treatment types have lower quality due to “inconsistent quality of creators,^[19]^” and etiology-related content exhibited limited volume with substantial quality variation due to high creation barriers and unmet market demand.^[31]^

Correlation analysis validated 2 major patterns: First, GQS is strongly correlated with mDISCERN, confirming the intrinsic link between “high quality and high reliability”^[23]^; Second, interaction indicators show strong correlation, reflecting the synergistic nature of user interactions,^[18]^ while interaction shows no correlation with quality (e.g., GQS and likes), indicating that platform traffic popularity does not reflect professional value and that public media literacy education needs to be strengthened.^[34]^

### 4.4. Limitations

This study has several limitations. First, the study only focused on 2 major domestic platforms, TikTok and Bilibili, excluding international short-video platforms such as YouTube and TikTok, which may limit the applicability of the research conclusions. Second, the study ultimately included 157 videos for analysis, and the data volume was relatively limited, perhaps unable to fully reflect the overall characteristics of muscle strain-related content in the short-video field. Finally, although the study reduced bias through standardized evaluation processes, randomization of video evaluation order, and the introduction of arbiters, the assessment of video quality and reliability still relied on the subjective judgment of researchers, inevitably introducing certain subjective bias. The two main evaluators in this study were graduate students majoring in sports rehabilitation with a research background in sports medicine. Although they were trained and pre-evaluated by a unified scale, compared with clinicians or senior sports medicine experts, their experience in the diagnosis and treatment of muscle strain and the interpretation of evidence-based guidance still has certain limitations, which may have a slight impact on the professional judgment of some video content.

## 5. Conclusions

This cross-sectional study systematically evaluated the quality and reliability of muscle strain-related short-form videos on Bilibili and TikTok. Bilibili demonstrated overall higher-quality muscle-strain-related short-video content with a unimodal score distribution, whereas TikTok showed a bimodal quality distribution and contained a larger proportion of low-quality videos. User engagement indicators on TikTok were markedly higher than those on Bilibili; however, engagement metrics were not correlated with objective content quality scores in the analyzed sample. Both platforms exhibited substantial structural imbalance in science popularization topics. Therapeutic-focused content dominated available short-video resources, while content explaining injury etiology remained severely underrepresented. These findings highlight an urgent need to optimize the online health information ecosystem for sports-injury-related short-video resources. The results provide practical references for the public to identify trustworthy muscle strain-related health information, guide platform administrators to strengthen content quality governance, and encourage medical-sports professionals to develop accessible, evidence-based popular science materials. Future work may explore several promising directions. Longitudinal monitoring is needed to track dynamic shifts in the quality of health science popularization videos on short-video platforms over time. Cross-cultural comparative studies could examine differences in platform ecosystems across geographic regions. Intervention research may also test whether quality-labeling frameworks and professional creator certification programs improve public access to credible online health information about sports injuries.

## Author contributions

**Supervision:** Xiaolin Yao.

**Visualization:** Wenling Gou.

**Software:** Yuwen Shangguan.

**Resources:** Xiaobo Xu.

**Funding acquisition:** Taojing Zhuang.

**Writing – original draft:** Wenqian Du, Cheng Chen.








