# Supplementary material for: The quality and reliability of short videos about muscle strain on Bilibili and TikTok: Cross-sectional study
Source: Medicine (Baltimore). 2026 Oct 16;105(42):e51099. doi: 10.1097/MD.0000000000051099 (PMC13681164; doi:10.1097/MD.0000000000051099)
Supplement: Supplementary file 1 [file medi-105-e51099-s001.docx]

**Appendix 1:** Classification of Professional and Non-Professional Individuals and Organizations

| classify | Specific category | feature |
| --- | --- | --- |
| professional person | rehabilitation doctor | With official medical/rehabilitation certification and a systematic medical background, we provide clinical-grade professional advice. |
|  | Certified Sports Blogger | Certified by the platform or authoritative sports institutions, focusing on the professional field of sports, and officially endorsed by the profession. |
| professional institute | institution | It has official industry certification, standardized organizational structure, and content output in line with professional standards. |
| Non-professional individual | patient | No official certification in the professional field, based on personal experience, no systematic professional theory support. |
|  | Master's and PhD in sports | Possessing a theoretical foundation in sports science without professional certification in practice or content creation, falling into the category of "having professional background but lacking certification". |
|  | Non-verified blogger | No platform or authoritative organization certification, sharing sports-related content based on personal interest and amateur experience. |
| Non-professional organization | rehabilitation agency | Official certification in the medical/rehabilitation industry, but no platform certification |
|  | Specialized clubs | Registered with sports authorities or associations, but not certified by the platform |
