# Supplementary material for: The quality and reliability of short videos about muscle strain on Bilibili and TikTok: Cross-sectional study
Source: Medicine (Baltimore). 2026 Oct 16;105(42):e51099. doi: 10.1097/MD.0000000000051099 (PMC13681164; doi:10.1097/MD.0000000000051099)
Supplement: Supplementary file 2 [file medi-105-e51099-s002.docx]

**Appendix 2:**Quality and reliability scores by platform

| Variables | Total (n = 157) | bilibili (n = 70) | TikTok (n = 87) | Statistic | *P* |
| --- | --- | --- | --- | --- | --- |
|  |  |  |  |  |  |
| Likes, M (Q₁, Q₃) | 321.00 (91.50, 1371.00) | 138.00 (31.00, 384.50) | 813.00 (263.00, 3124.50) | Z=-5.82 | <.001 |
| Favorites, M (Q₁, Q₃) | 171.00 (51.00, 647.00) | 119.50 (38.50, 423.50) | 199.00 (69.00, 1021.50) | Z=-2.48 | 0.013 |
| Comments, M (Q₁, Q₃) | 25.00 (5.00, 123.00) | 11.00 (2.00, 49.00) | 58.00 (10.50, 205.00) | Z=-4.21 | <.001 |
| Shares, M (Q₁, Q₃) | 96.00 (22.00, 445.00) | 45.50 (5.00, 114.50) | 261.00 (52.00, 1150.50) | Z=-5.68 | <.001 |
| Video Duration, M (Q₁, Q₃) | 0.06 (0.04, 0.12) | 0.12 (0.08, 0.29) | 0.04 (0.03, 0.07) | Z=-6.02 | <.001 |
| Publish Time, M (Q₁, Q₃) | 45432.72 (44954.78, 45800.79) | 45407.78 (44963.18, 45731.46) | 45443.33 (44954.59, 45801.10) | Z=-0.11 | 0.909 |
| GQS, n(%) |  |  |  | χ²=12.74 | 0.013 |
| 1 | 16 (10.19) | 10 (14.29) | 6 (6.90) |  |  |
| 2 | 42 (26.75) | 12 (17.14) | 30 (34.48) |  |  |
| 3 | 69 (43.95) | 34 (48.57) | 35 (40.23) |  |  |
| 4 | 23 (14.65) | 8 (11.43) | 15 (17.24) |  |  |
| 5 | 7 (4.46) | 6 (8.57) | 1 (1.15) |  |  |
| DIS, n(%) |  |  |  | - | <.001 |
| 0 | 9 (5.73) | 9 (12.86) | 0 (0.00) |  |  |
| 1 | 20 (12.74) | 4 (5.71) | 16 (18.39) |  |  |
| 2 | 47 (29.94) | 14 (20.00) | 33 (37.93) |  |  |
| 3 | 48 (30.57) | 24 (34.29) | 24 (27.59) |  |  |
| 4 | 26 (16.56) | 12 (17.14) | 14 (16.09) |  |  |
| 5 | 7 (4.46) | 7 (10.00) | 0 (0.00) |  |  |
