# Supplementary material for: The quality and reliability of short videos about muscle strain on Bilibili and TikTok: Cross-sectional study
Source: Medicine (Baltimore). 2026 Oct 16;105(42):e51099. doi: 10.1097/MD.0000000000051099 (PMC13681164; doi:10.1097/MD.0000000000051099)
Supplement: Supplementary file 3 [file medi-105-e51099-s003.docx]

**Appendix 3:**Quality and reliability scores by creator source

| Variables | Total (n = 157) | Non-professional individuals (n = 38) | Non-professional institutions (n = 60) | Professional individuals (n = 50) | Professional institutions (n = 9) | Statistic | *P* |
| --- | --- | --- | --- | --- | --- | --- | --- |
|  |  |  |  |  |  |  |  |
| GQS, n(%) |  |  |  |  |  | - | 0.048* |
| 1.00 | 16 (10.19) | 9 (23.68) | 4 (6.67) | 3 (6.00) | 0 (0.00) |  |  |
| 2.00 | 42 (26.75) | 6 (15.79) | 18 (30.00) | 15 (30.00) | 3 (33.33) |  |  |
| 3.00 | 69 (43.95) | 14 (36.84) | 25 (41.67) | 26 (52.00) | 4 (44.44) |  |  |
| 4.00 | 23 (14.65) | 4 (10.53) | 11 (18.33) | 6 (12.00) | 2 (22.22) |  |  |
| 5.00 | 7 (4.46) | 5 (13.16) | 2 (3.33) | 0 (0.00) | 0 (0.00) |  |  |
| Total of DSI, n (%) |  |  |  |  |  | - | 0.207* |
| 0.00 | 9 (5.73) | 5 (13.16) | 2 (3.33) | 2 (4.00) | 0 (0.00) |  |  |
| 1.00 | 20 (12.74) | 6 (15.79) | 9 (15.00) | 5 (10.00) | 0 (0.00) |  |  |
| 2.00 | 47 (29.94) | 6 (15.79) | 18 (30.00) | 19 (38.00) | 4 (44.44) |  |  |
| 3.00 | 48 (30.57) | 10 (26.32) | 19 (31.67) | 16 (32.00) | 3 (33.33) |  |  |
| 4.00 | 26 (16.56) | 6 (15.79) | 10 (16.67) | 8 (16.00) | 2 (22.22) |  |  |
| 5.00 | 7 (4.46) | 5 (13.16) | 2 (3.33) | 0 (0.00) | 0 (0.00) |  |  |
| -: Fisher exact, *: Simulated p-value | | | | | | | |
