# Supplementary material for: The quality and reliability of short videos about muscle strain on Bilibili and TikTok: Cross-sectional study
Source: Medicine (Baltimore). 2026 Oct 16;105(42):e51099. doi: 10.1097/MD.0000000000051099 (PMC13681164; doi:10.1097/MD.0000000000051099)
Supplement: Supplementary file 4 [file medi-105-e51099-s004.docx]

**Appendix 4:**Quality and reliability scores by video theme

| Variables | Total (n = 157) | Cause (n = 6) | Scientific Introduction (n = 9) | Judge (n = 12) | Treatment (n = 130) | Statistic | *P* |
| --- | --- | --- | --- | --- | --- | --- | --- |
|  |  |  |  |  |  |  |  |
| GQS, n(%) |  |  |  |  |  | - | 0.420 |
| 1.00 | 16 (10.19) | 0 (0.00) | 0 (0.00) | 0 (0.00) | 16 (12.31) |  |  |
| 2.00 | 42 (26.75) | 1 (16.67) | 0 (0.00) | 6 (50.00) | 35 (26.92) |  |  |
| 3.00 | 69 (43.95) | 4 (66.67) | 7 (77.78) | 5 (41.67) | 53 (40.77) |  |  |
| 4.00 | 23 (14.65) | 1 (16.67) | 2 (22.22) | 1 (8.33) | 19 (14.62) |  |  |
| 5.00 | 7 (4.46) | 0 (0.00) | 0 (0.00) | 0 (0.00) | 7 (5.38) |  |  |
| DIS, n(%) |  |  |  |  |  | - | 0.194* |
| 0.00 | 9 (5.73) | 0 (0.00) | 0 (0.00) | 0 (0.00) | 9 (6.92) |  |  |
| 1.00 | 20 (12.74) | 0 (0.00) | 0 (0.00) | 3 (25.00) | 17 (13.08) |  |  |
| 2.00 | 47 (29.94) | 1 (16.67) | 0 (0.00) | 5 (41.67) | 41 (31.54) |  |  |
| 3.00 | 48 (30.57) | 4 (66.67) | 6 (66.67) | 2 (16.67) | 36 (27.69) |  |  |
| 4.00 | 26 (16.56) | 1 (16.67) | 3 (33.33) | 1 (8.33) | 21 (16.15) |  |  |
| 5.00 | 7 (4.46) | 0 (0.00) | 0 (0.00) | 1 (8.33) | 6 (4.62) |  |  |
| -: Fisher exact, *: Simulated p-value | | | | | | | |
